# l-Lactate treatment by photosynthetic cyanobacteria expressing heterogeneous l-lactate dehydrogenase

**DOI:** 10.1038/s41598-023-34289-3

**Published:** 2023-05-04

**Authors:** Yuichi Kato, Kosuke Inabe, Yuji Haraguchi, Tatsuya Shimizu, Akihiko Kondo, Tomohisa Hasunuma

**Affiliations:** 1 Engineering Biology Research Center, Kobe University, 1-1 Rokkodai, Nada, Kobe, 657-8501 Japan; 2 Graduate School of Science, Technology and Innovation, Kobe University, 1-1 Rokkodai, Nada, Kobe, 657-8501 Japan; 3 Institute of Advanced Biomedical Engineering and Science, TWIns, Tokyo Women’s Medical University, 8-1 Kawada, Shinjuku, Tokyo 162-8666 Japan; 4 Department of Chemical Science and Engineering, Graduate School of Engineering, Kobe University, 1-1 Rokkodai, Nada, Kobe, 657-8501 Japan

**Keywords:** Biotechnology, Microbiology, Plant sciences

## Abstract

l-Lactate is a major waste compound in cultured animal cells. To develop a sustainable animal cell culture system, we aimed to study the consumption of l-lactate using a photosynthetic microorganism. As genes involved in l-lactate utilization were not found in most cyanobacteria and microalgae, we introduced the NAD-independent l-lactate dehydrogenase gene from *Escherichia coli* (*lldD*) into *Synechococcus* sp. PCC 7002. The *lldD*-expressing strain consumed l-lactate added to basal medium. This consumption was accelerated by expression of a lactate permease gene from *E. coli* (*lldP*) and an increase in culture temperature. Intracellular levels of acetyl-CoA, citrate, 2-oxoglutarate, succinate, and malate, and extracellular levels of 2-oxoglutarate, succinate, and malate, increased during l-lactate utilization, suggesting that the metabolic flux from l-lactate was distributed toward the tricarboxylic acid cycle. This study provides a perspective on l-lactate treatment by photosynthetic microorganisms, which would increase the feasibility of animal cell culture industries.

## Introduction

**Figure 1:**
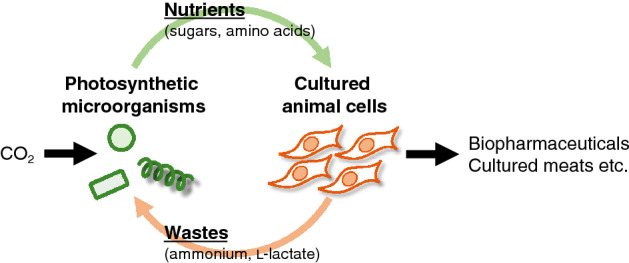
Scheme of circular cell culture (CCC).

Cultured animal cells are valuable in industries such as biopharmaceutical production^1,2^. Cultured meat production has also been studied, in which only edible parts are produced by cultured animal cells^3,4^. To produce nutrients for animal cells (e.g. sugars and amino acids), photosynthetic microorganisms (i.e. prokaryotic cyanobacteria and eukaryotic microalgae) have been recently studied because of their high ability to produce biomass from atmospheric CO_2_^5^. Mouse C2C12 myoblasts and primary bovine myoblasts have been successfully cultured using cell extracts from *Chlorella vulgaris*, *Chlorococcum littorale*, and *Arthrospira platensis* as nutrients^6,7^. Thus, by treating waste compounds from animal cells (such as ammonium and l-lactate) using a photosynthetic microorganism, a sustainable animal cell culture system, that is, a circular cell culture (CCC) system, can be developed^8^ (Fig. 1). Consumption of ammonium in the culture waste of C2C12 cells has already been achieved through the cultivation of *C. vulgaris* and *C. littorale*^9^. The potential application of the CCC system was previously shown using *C. littorale*, RL34 hepatocytes, and C2C12 myoblasts as producers of nutrients, growth factors, and muscles, respectively^8^. However, l-lactate consumption by photosynthetic microorganisms has not yet been investigated. l-Lactate is a major waste compound in cultured animal cells, and its accumulation in medium causes cytotoxic effects by changing the pH and osmolarity^10^. Therefore, l-lactate removal is a ubiquitous requirement in industries that utilize cultured animal cells and is necessary for optimal functioning of the CCC system.

**Figure 2:**
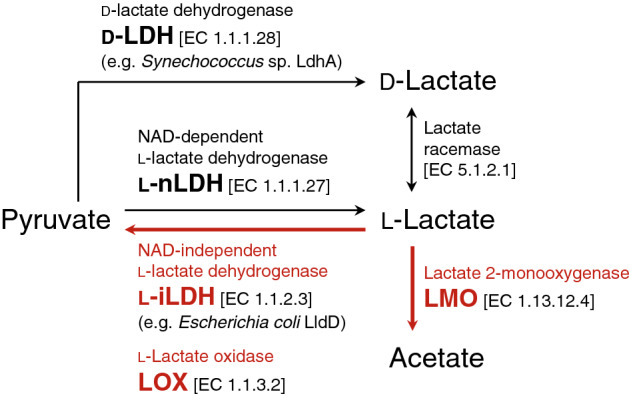
Enzymes involved in l-lactate metabolism.

Cyanobacteria such as *Synechococcus* sp. PCC 7002 and *Synechocystis* sp. PCC 6803 harbor d-lactate dehydrogenase (d-LDH; EC 1.1.1.28) as an enzyme involved in lactate metabolism and can produce d-lactate^11,12^ (Fig. 2). The genetically engineered strains of PCC 6803 into which NAD-dependent l-lactate dehydrogenase (l-nLDH, EC 1.1.1.27) has been introduced can also produce l-lactate^13^. However, the ability of microalgae and cyanobacteria to consume l-/d-lactate remains unclear. Several heterotrophic bacteria, such as *Escherichia coli*, *Corynebacterium glutamicum*, and *Pseudomonas aeruginosa*, can utilize l-lactate with the help of NAD-independent l-lactate dehydrogenase (l-iLDH, EC 1.1.2.3), which catalyzes the conversion of l-lactate into pyruvate^14–16^. For example, *E. coli* harbors the lactate operon, which is composed of the *lldD* (l-iLDH) and *lldP* (lactate permease) genes for l-lactate utilization as well as the *lldR* (regulatory protein) gene^14^.

The present study aimed to develop a method for l-lactate consumption using a photosynthetic microorganism. Most cyanobacteria and microalgae do not possess genes for l-lactate metabolism and cannot utilize l-lactate. Therefore, the ability for l-lactate utilization was added to PCC 7002 by heterogeneous expression of the *lldD* and *lldP* genes from *E. coli*. The distribution of metabolic flux derived from l-lactate was determined through metabolome analysis. This study provides a perspective on l-lactate treatment by photosynthetic microorganisms and establishes a method to develop cyanobacteria with the ability to utilize l-lactate. These findings will be valuable to industries using animal cell cultures and would increase the feasibility of the CCC system.

## Results and discussion

### l-Lactate utilization ability of cyanobacteria and microalgae in nature

l-Lactate has cytotoxic effects on animal cells^10^. In the present study, C2C12 cells were examined, and addition of l-lactate higher than 20 mM significantly decreased cell viability (Supplementary Fig. 1). To achieve a sustainable CCC system^8^ (Fig. 1), we aimed to develop a method for l-lactate consumption using a photosynthetic microorganism. The conservation of genes involved in l-lactate metabolism was first investigated in silico to examine the l-lactate utilization ability of photosynthetic microorganisms in nature (Fig. 2). In this investigation, cyanobacteria and microalgae harboring l-iLDH (EC 1.1.2.3), l-lactate oxidase (LOX, EC 1.1.3.2), or lactate 2-monoxygenase (LMO, EC 1.13.12.4) genes were identified using the Kyoto Encyclopedia of Genes and Genomes (KEGG) database^17^. l-iLDH and LOX convert l-lactate into pyruvate, and LMO converts l-lactate into acetate^18^. In contrast, l-nLDH (EC 1.1.1.27) synthesizes l-lactate from pyruvate reversibly or irreversibly^19^. Therefore, cyanobacteria and microalgae harboring LOX and LMO genes were not found. In addition, the l-iLDH gene is absent in most cyanobacteria and microalgae, except *Aureococcus anophagefferens*^20^, *Crocosphaera watsonii*^21^, *Trichodesmium erythraeum*^22^, and *Rivularia* sp.^23^, among which *A. anophagefferens* and *T. erythraeum* are known to cause harmful algal blooms. Thus, the present study found that l-lactate utilization genes are absent in most cyanobacteria and microalgae.

To experimentally examine the l-lactate utilization ability, several cyanobacteria and microalgae, i.e. *Synechococcus* sp. PCC 7002^24^, *Anabaena* sp. PCC 7120^25^, *Arthrospira platensis* NIES-39^26^, *Chlamydomonas* sp. KOR1^27^, and *Pavlova* sp. OPMS 30543^28^, were cultured phototrophically in the presence of l-lactate. The growth of these cyanobacteria and microalgae, except for PCC 7120, was not suppressed by 20 mM l-lactate, which is nearly equivalent to the culture waste of animal cells. However, lactate concentration in the medium did not change during cultivation (Supplementary Fig. 2). These results suggest that few cyanobacteria and microalgae species in nature can utilize l-lactate. Therefore, genetic engineering should be a suitable approach for creating photosynthetic microorganisms capable of utilizing l-lactate.

### Genetic engineering of cyanobacteria for l-lactate utilization

**Figure 3:**
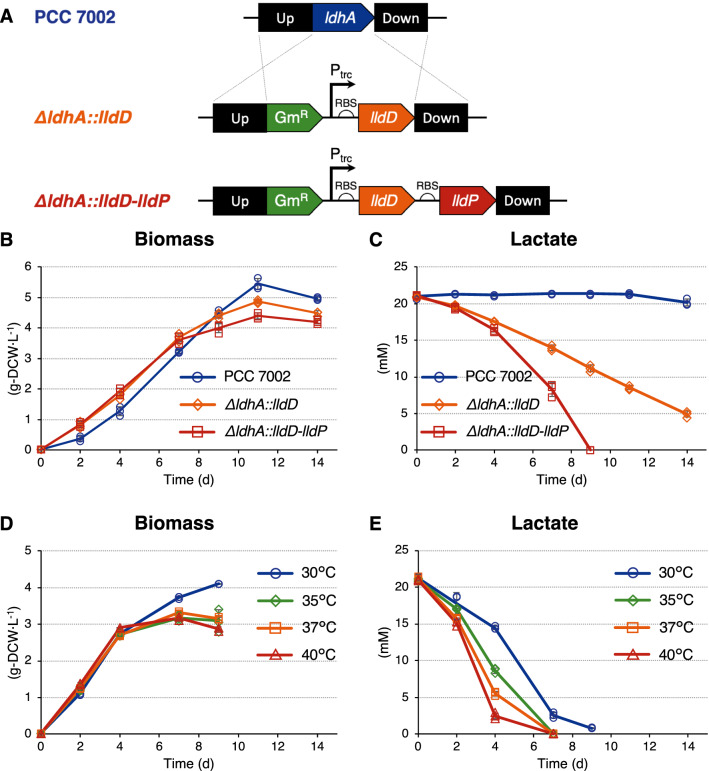
Evaluation of the *lldD*/*lldP*-expressing cyanobacteria. (**A**) Genetic elements for l-lactate utilization. The *ldhA* (d-lactate dehydrogenase) gene in the pAQ7 plasmid of *Synechococcus* sp. PCC 7002 was replaced with the gentamicin resistance cassette (Gm^R^) and codon-optimized NAD-independent l-lactate dehydrogenase (*lldD*) and lactate permease (*lldP*) genes from *Escherichia coli*. The *lldD* and *lldP* genes were constitutively expressed in the *trc* promoter region. The black boxes indicate homologous sequences used to introduce these genetic elements into the *ldhA* gene site. (**B**,**C**) Comparison of the *lldD*/*lldP*-expressing strains. Cyanobacteria were phototrophically cultured in the presence of 20 mM l-lactate, and the dry cell weight (DCW)-based biomass concentrations (**B**) and lactate concentration in the medium (**C**) were investigated. (**D**,**E**) Influence of temperature on l-lactate utilization. The cyanobacteria expressing both *lldD* and *lldP* were phototrophically cultured in the presence of 20 mM l-lactate at 30–40 °C, and DCW-based biomass concentrations (**D**) and lactate concentration in the medium (**E**) were investigated. Error bars indicate standard deviation of three replicate experiments.

To develop photosynthetic microorganisms capable of l-lactate utilization, this study introduced heterogenous genes into the marine cyanobacterium PCC 7002, which is tolerant to salinity of animal medium. Because several heterotrophic bacteria can grow using l-lactate due to l-iLDH^14–16^, the present study employed l-iLDH encoded by the *E. coli lldD* gene. The *lldP* gene encoding lactate permease was previously shown to improve d-lactate export in cyanobacteria such as PCC 7002 and *Synechococcus elongatus* PCC 7942^12,29,30^. This study employed the *lldP* gene to enhance l-lactate uptake. The *lldD* and *lldP* genes were expressed in PCC 7002 cells using the constitutive *trc* promoter (Fig. 3A). These genetic elements were introduced into the *ldhA* gene site encoding d-LDH to prevent d-lactate synthesis from pyruvate by the native enzyme. Integration and complete segregation were confirmed using PCR (Supplementary Fig. 3).

To evaluate the l-lactate utilization ability, PCC 7002 and the recombinant strains were phototrophically cultured in the presence of 20 mM l-lactate. During the early stage of cultivation, the recombinant strains showed more enhanced cell growth than PCC 7002 (Fig. 3B). l-lactate concentration in the medium significantly decreased in the *lldD*-expressing strain, while it remained unchanged in PCC 7002 (Fig. 3C). Additional introduction of the *lldP* gene enhanced l-lactate consumption by the *lldD*-expressing strain, and as a result, 20 mM (1.8 g·L^−1^) of l-lactate was completely consumed in 9 days. Thus, the l-lactate utilization ability was successfully added to PCC 7002 using the *lldD*/*lldP* genes. These results indicate that both LldD and LldP proteins were functional in cyanobacteria and that *lldP* can contribute to l-lactate import.

To accelerate l-lactate consumption, the culture temperature of the *lldD*/*lldP*-expressing strain was examined. During the initial 4 days, biomass concentration was not significantly different at 30–40 °C (Fig. 3D). In contrast, higher temperatures resulted in higher l-lactate consumption, and 20 mM l-lactate was completely consumed in 7 days at 35–40 °C (Fig. 3E). This result indicates that l-lactate utilization by the *lldD*/*lldP*-expressing strain can be enhanced by elevated temperatures probably because this temperature range is suitable for these *E. coli* enzymes. Thus, we established a method to develop cyanobacteria with the l-lactate utilization ability. Addition of a functional l-iLDH would also be valuable in adding l-lactate utilization ability to microalgae.

### Distribution of metabolic flux from l-lactate

**Figure 4:**
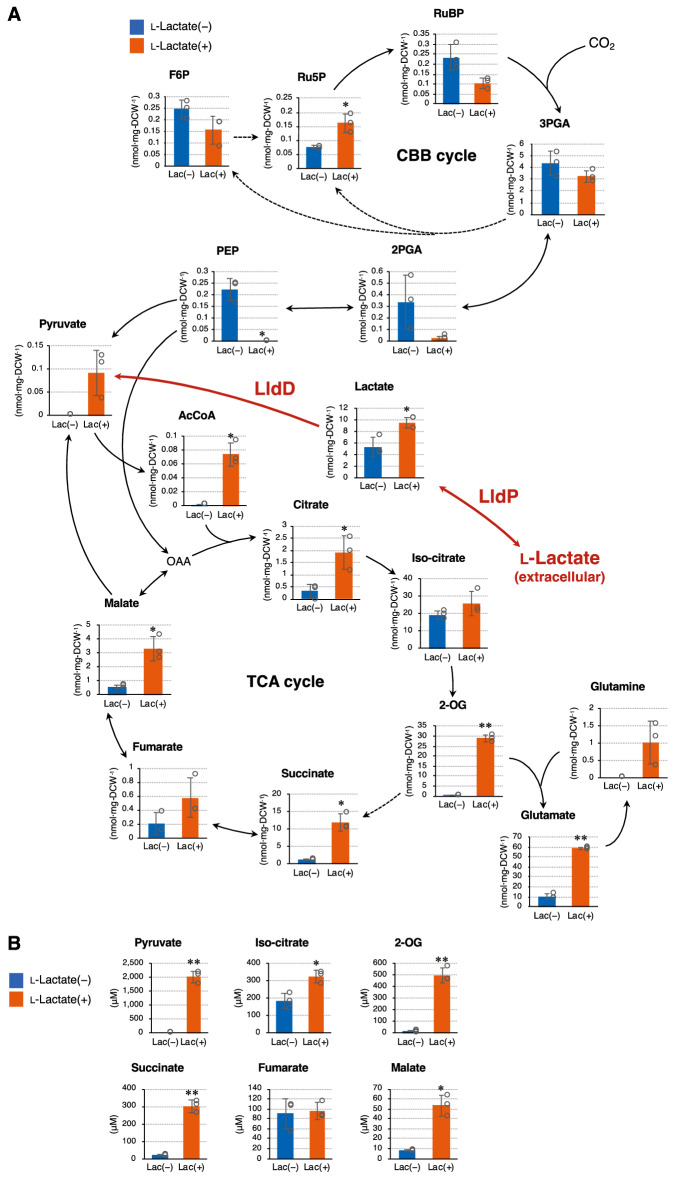
Metabolome analysis during l-lactate utilization. The *lldD*/*lldP*-expressing strain was phototrophically cultured in the absence and presence of 20 mM l-lactate at 30 °C for 7 days. (**A**) Intracellular metabolites in *lldD*/*lldP*-expressing cells. The solid and dotted lines represent single and multiple enzymatic steps, respectively. (**B**) Extracellularly released metabolites from *lldD*/*lldP*-expressing cells. Error bars indicate the standard deviation of three replicate experiments (*P < 0.05, **P < 0.01, Welch’s *t* test). *2-OG* 2-oxoglutarate, *2-PGA* 2-phosphoglyceric acid, *3-PGA* 3-phosphoglyceric acid, *AcCoA* acetyl-CoA, *CBB cycle* Calvin–Benson–Bassham cycle, *DCW* dry cell weight, *F6P* fructose 6-phosphate, *PEP* phosphoenolpyruvate. *Ru5P* ribulose-5-phosphate, *RuBP* ribulose-1,5-bisphosphate, *TCA cycle* tricarboxylic acid cycle.

In *lldD/lldP*-expressing cells, pyruvate synthesized by LldD is converted to other metabolites by intrinsic enzymes. To elucidate the distribution of the metabolic flux derived from l-lactate, metabolome analysis of *lldD*/*lldP*-expressing cells cultured in the absence and presence of l-lactate was performed. Lactate, pyruvate, and acetyl-CoA (AcCoA) accumulated in cells when supplemented with l-lactate (Fig. 4A). In addition, several metabolites of the tricarboxylic acid (TCA) cycle, that is, citrate, 2-oxoglutarate (2-OG), succinate, and malate, accumulated in the presence of l-lactate. These results indicate that the metabolic flux from l-lactate was largely distributed to the TCA cycle.

A significant intracellular accumulation of these metabolites should trigger their releases from *lldD*/*lldP*-expressing cells during l-lactate utilization. By analyzing the metabolites in the culture supernatant, we found that several metabolites increased extracellularly when supplemented with l-lactate (Fig. 4B). Extracellular levels of pyruvate, 2-OG, succinate, and malate in the *lldD*/*lldP*-expressing strain when supplemented with 20 mM l-lactate were 2,021.1, 496.7, 300.6, and 53.3 μM, respectively. The accumulation of intracellular metabolites by l-lactate utilization likely caused a significant release of these organic acids. A similar phenomenon, with elevated release of organic acids such as pyruvate and 2-OG, has been reported in glycogen-deficient cyanobacterial mutants^31–33^. Because 2-OG can be converted into glutamine, which is an essential and abundant amino acid in the medium for mammalian cell cultures^34^, the *lldD*/*lldP*-expressing strain may also be valuable for producing amino acids in animal cells.

### Photosynthetic activity during l-lactate utilization

**Figure 5:**
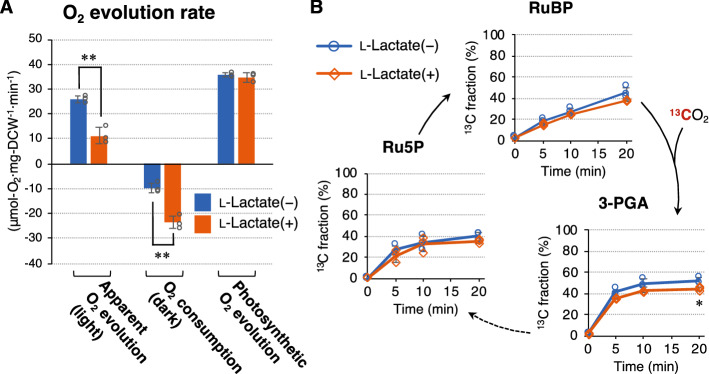
Photosynthetic activity during l-lactate utilization. *lldD/lldP*-expressing cells were phototrophically cultured in the absence and presence of 20 mM l-lactate at 30 °C for 3 days and then subjected to analyses of photosynthetic activity. (**A**) Oxygen evolution rate. The photosynthetic O_2_ evolution rate was calculated by subtracting the O_2_ consumption rate (dark) from the apparent O_2_ evolution rate (light). (**B**) ^13^C fraction of metabolites in the Calvin–Benson–Bassham (CBB) cycle. Newly synthesized metabolites from CO_2_ were ^13^C-labeled by incubating the cells with NaH^13^CO_3_ as the carbon source. Error bars indicate the standard deviation of three replicate experiments (*P < 0.05, **P < 0.01, Welch’s *t* test). *3-PGA* 3-phosphoglyceric acid, *DCW* dried cell weight, *Ru5P* ribulose-5-phosphate, *RuBP* ribulose-1,5-bisphosphate.

To examine whether the photosynthetic activity of *lldD/lldP*-expressing cells was affected by l-lactate utilization, the cells were analyzed in the presence and absence of l-lactate. First, O_2_ evolution was analyzed on day 3 when the cells performed biomass production and l-lactate utilization (Fig. 3). The apparent O_2_ evolution rate measured under light conditions significantly decreased in the presence of l-lactate (Fig. 5A). The O_2_ consumption rate was measured in the dark and was found to be significantly increased when l-lactate was supplied to the medium. This might be due to the enhanced metabolic flux of the TCA cycle during l-lactate utilization (Fig. 4A) as the TCA cycle is the dominant source of NAD(P)H for respiratory electron transport^35^. The net photosynthetic O_2_ evolution rate was calculated by subtracting the O_2_ consumption rate from the apparent O_2_ evolution rate and was found to be almost the same in the presence or absence of l-lactate.

To further examine photosynthetic activity, the CO_2_ fixation activity of *lldD/lldP*-expressing cells was analyzed by in vivo ^13^C labeling experiments. NaH^13^CO_3_ was added to the medium on day 3 to supply the *lldD/lldP*-expressing cells with ^13^CO_2_. After incubation for 0–20 min, the ^13^C fraction of metabolites in the Calvin–Benson–Bassham (CBB) cycle, which conducts photosynthetic CO_2_ fixation by ribulose 1,5-bisphosphate carboxylase/oxygenase (Rubisco), was analyzed. The de novo synthesis of 3-phosphoglyceric acid (3-PGA), ribulose-5-phosphate (Ru5P), and ribulose-1,5-bisphosphate (RuBP) from CO_2_ occurred even in the presence of l-lactate, although the ^13^C fraction of 3-PGA was slightly decreased (Fig. 5B). These results reveal that photosynthesis in the *lldD/lldP*-expressing cells continued even during l-lactate utilization. In the presence of l-lactate, the de novo synthesis of Ru5P was not changed (Fig. 5B), while its accumulation significantly increased (Fig. 4A). These results are contrasting but not necessarily conflicting because these experiments analyzed for distinct metabolic parameters at different time points. The higher growth rate of l-lactate-assimilating cyanobacteria than that of PCC 7002 (Fig. 3B) might be due to the simultaneous utilization of CO_2_ and l-lactate as carbon sources. Thus, this study established a method to consume l-lactate in animal cell cultures using photosynthetic cyanobacteria expressing the heterogenous *lldD* and *lldP* genes. The findings of this study will contribute to the development of a sustainable CCC system for the animal cell culture industry.

## Materials and methods

### Strains and culture conditions

Cyanobacterium *Synechococcus* sp. PCC 7002 and the recombinant strains were phototrophically cultured in double-deck flasks on a BR-40LF bioshaker (TAITEC, Aichi, Japan). The upper stage of the flasks was supplemented with 70 mL of Medium A2 (8.30 × 10^–3^ M tris(hydroxymethyl)aminomethane, 1.76 × 10^–2^ M NaNO_3_, 3.10 × 10^–1^ M NaCl, 2.00 × 10^–2^ M MgSO_4_·7H_2_O, 2.50 × 10^–3^ M CaCl_2_·2H_2_O, 3.70 × 10^–4^ M KH_2_PO_4_, 8.10 × 10^–3^ M KCl, 8.90 × 10^–5^ M Na_2_EDTA·2H_2_O, 3.00 × 10^–5^ M FeCl_3_·6H_2_O, 5.50 × 10^–4^ M H_3_BO_3_, 2.20 × 10^–5^ M MnCl_2_·4H_2_O, 2.30 × 10^–6^ M ZnCl_2_, 2.10 × 10^–7^ M Na_2_MoO_4_·2H_2_O, 1.20 × 10^–8^ M CuSO_4_·5H_2_O, 5.10 × 10^–8^ M CoCl_2_·6H_2_O, and 3.00 × 10^–9^ M vitamin B_12_), containing 40 mg·L^−1^ gentamicin when necessary. To investigate l-lactate utilization, 20 mM l-lactate (Sigma-Aldrich, St. Louis, MO, USA) and 20 mM NaOH were added to the medium. The lower stage of the flasks was supplemented with 50 mL of 2 M K_2_CO_3_/KHCO_3_ solution, which adjusted the CO_2_ gas concentration to 2% (*v/v*). Cells were inoculated at an optical density of 750 nm (OD_750_) = 0.1 and cultured under continuous illumination with white fluorescent lamps at 100 µmol photons·m^−2^·s^−1^ at 30 °C with rotary shaking at 100 rpm^24^.

### Construction of recombinant strains

The pUC118-based vectors, harboring the *trc* promoter and homology arms for the *ldhA* gene (SYNPCC7002_G0164) in the pAQ7 plasmid of *Synechococcus* sp., were used to introduce the *lldD* and *lldP* genes from *E. coli* via homologous recombination. The codon-optimized genes of LldD (NP_418062.1) and LldP (NP_418060.1) were prepared by the Genscript gene synthesis service and cloned into the vector using the In-Fusion HD Cloning Kit (Takara Bio USA, Inc., Mountain View, CA, USA). PCC 7002 was transformed as previously described^24^. Integration and complete segregation were confirmed by PCR using the specific primer pair 5′-AGACATTTCCCACAGACCACATCAAATTA-3′ and 5′-GGATCAATTTACGTCTTTGTTGGCGCA-3′.

### Measurement of lactate

Each culture was centrifuged at 8000×*g* for 5 min. The supernatant was filtered using a Shim-pack SPR-Pb column (Shimadzu, Kyoto, Japan) and analyzed using a high-performance liquid chromatography system (Shimadzu) equipped with an Aminex HPX-87H column (Bio-Rad Laboratories, Hercules, CA, USA). l-Lactate (Sigma-Aldrich) was used as the quantitative standard to determine the lactate concentration using a calibration curve.

### Metabolome analysis

To prepare intracellular metabolites, a culture broth containing cells equivalent to 5 mg dry weight was mixed with four times the volume of 32.5% (*v/v*) methanol pre-cooled at − 30 °C. The mixture was centrifuged at 8000×*g* for 3 min at 4 °C. After complete removal of the supernatant, the cells were washed with 20 mM ammonium carbonate once and immediately resuspended in 1 mL of pre-cooled methanol containing 37.5 μM l-methionine sulfone and 37.5 μM piperazine-1,4-bis(2-ethanesulfonic acid) (PIPES) as internal standards. The cell suspension (500 μL) was added to 200 μL ultrapure water and 500 μL chloroform pre-cooled at 4 °C and then vigorously mixed using vortexing for 30 s. After centrifugation at 14,000×*g* for 5 min at 4 °C, the aqueous layer was collected and filtered using an Amicon Ultra-0.5 Centrifugal Filter Unit UFC5003BK (Merck Millipore, Burlington, MA, USA) by centrifugation at 14,000×*g* at 4 °C. The sample (300 μL) was dried under vacuum using a centrifugal evaporator CEV-3100 (EYELA, Tokyo, Japan) and resuspended in 20 µL of ultrapure water. To prepare extracellular metabolites, the culture was centrifuged at 8000×*g* for 5 min. The supernatant (500 μL) was mixed with 500 μL chloroform pre-cooled at 4 °C by vortexing. After centrifugation at 14,000×*g* for 5 min at 4 °C, the upper layer was collected and filtered using UFC5003BK (Merck Millipore) as described above. Next, 400 μM l-methionine sulfone and 400 μM PIPES were added as internal standards. The intracellular and extracellular samples were subjected to capillary electrophoresis time-of-flight mass spectrometry (CE-TOFMS) using a G7100 CE and G6224AA liquid chromatography-mass selective detector (LC/MSD) TOF system (Agilent Technologies, Santa Clara, CA, USA)^24^.

### Analysis of photosynthesis

Cells were cultivated for 3 days under the conditions described above. To analyze the O_2_ evolution rate, cultured cells were harvested, centrifuged at 8000×*g* for 5 min, and resuspended in fresh Medium A2 with or without 20 mM l-lactate to adjust the cell density to OD_750_ = 5.0. The O_2_ concentration in the cell suspension was measured using an oxygen electrode (Hansatech, King’s Lynn, UK). During the measurements, the cell suspension was maintained at 30 °C and mixed using a magnetically controlled microstirrer. The O_2_ consumption rate of the cells was determined by measuring the O_2_ concentration in the dark, while the apparent O_2_ evolution rate was determined under illumination with a red LED light (200 μmol photons·m^−2^·s^−1^). The photosynthetic O_2_ evolution rate was calculated by subtracting the O_2_ consumption rate from the apparent O_2_ evolution rate^24^.

To investigate carbon fixation from CO_2_, in vivo ^13^C labeling of metabolites in the CBB cycle was performed. After 3 d of cultivation, the cell culture was added to 25 mM NaH^13^CO_3_ as a carbon source and incubated under illumination with white fluorescent lamps (100 μmol photons·m^−2^·s^−1^). After labeling for 0–20 min, the intracellular metabolites were analyzed as described above^24^. The ratio of ^13^C in the total carbon (^13^C fraction) of the metabolite was determined based on the shifts between the ^12^C and ^13^C mass spectra.

### Statistics and reproducibility

Data in this study are represented as mean ± standard deviation of three replicate experiments. Statistical significance was determined by Welch’s *t* test.

## Supplementary Information

Supplementary Figures.

## Data Availability

The data supporting the findings of this study are available from the corresponding author upon reasonable request.
