# Supplementary material for: l-Lactate treatment by photosynthetic cyanobacteria expressing heterogeneous l-lactate dehydrogenase
Source: Sci Rep. 2023 May 4;13:7249. doi: 10.1038/s41598-023-34289-3 (PMC10160077; doi:10.1038/s41598-023-34289-3)
Supplement: Supplementary file 1 — Supplementary Figures. [file 41598_2023_34289_MOESM1_ESM.pdf]

Supplementary information

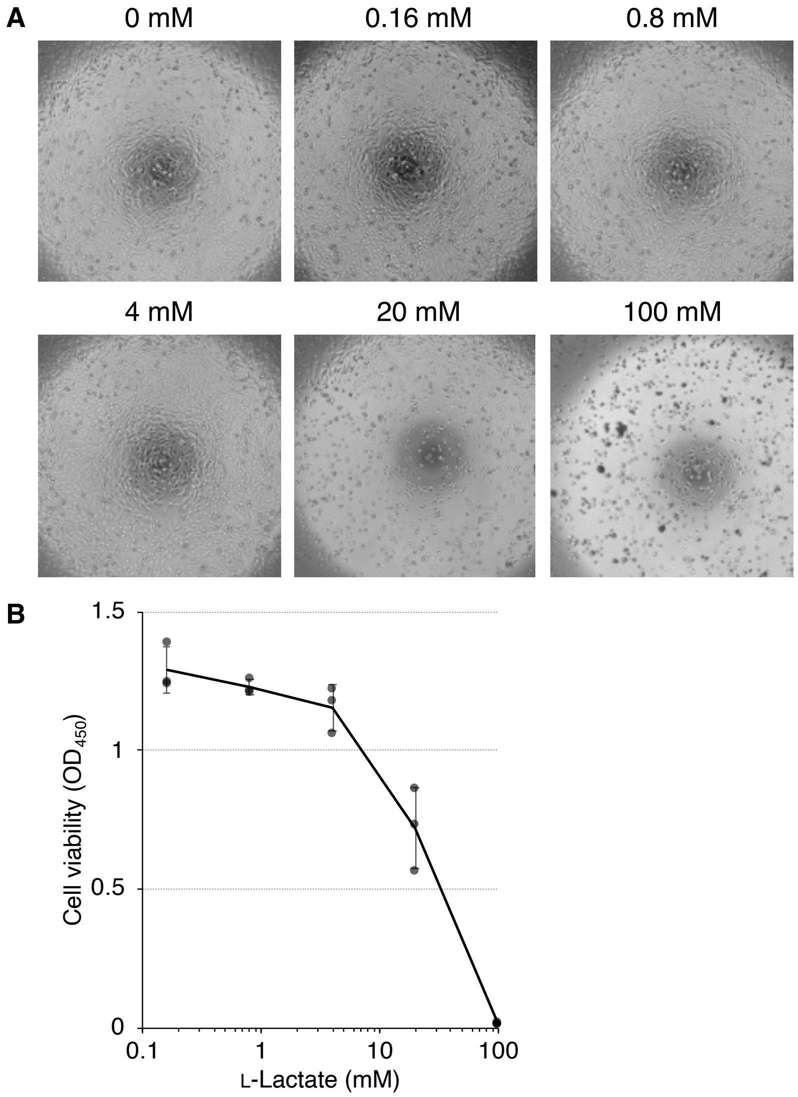

Supplementary Figure 1. Cytotoxic effect of L-lactate on mouse C2C12 cells. C2C12 cells (ATCC® CRL-1772™) cultured in a conventional animal cell culture medium (Dulbecco's Modified Eagle Medium [Sigma-Aldrich, St. Louis, MO, USA] supplemented with 10% fetal bovine serum [Japan Bio Serum, Nagoya, Japan] and 1% penicillin/streptomycin [Invitrogen, Carlsbad, CA, USA]) were incubated with 0–100 mM L-lactate at 37 °C for 16 h under a humidified atmosphere with 5% CO_2. After incubation, the cytotoxic effect of L-lactate was assessed by microscopy (ECLIPSE TS2, Nikon, Tokyo, Japan) using a software (NIS-Elements BR, Nikon) (A) and a commercially available XTT assay kit (XTT assay, Biological Industries, Cromwell, CT, USA) (B). The XTT assay was performed as previously described[6].

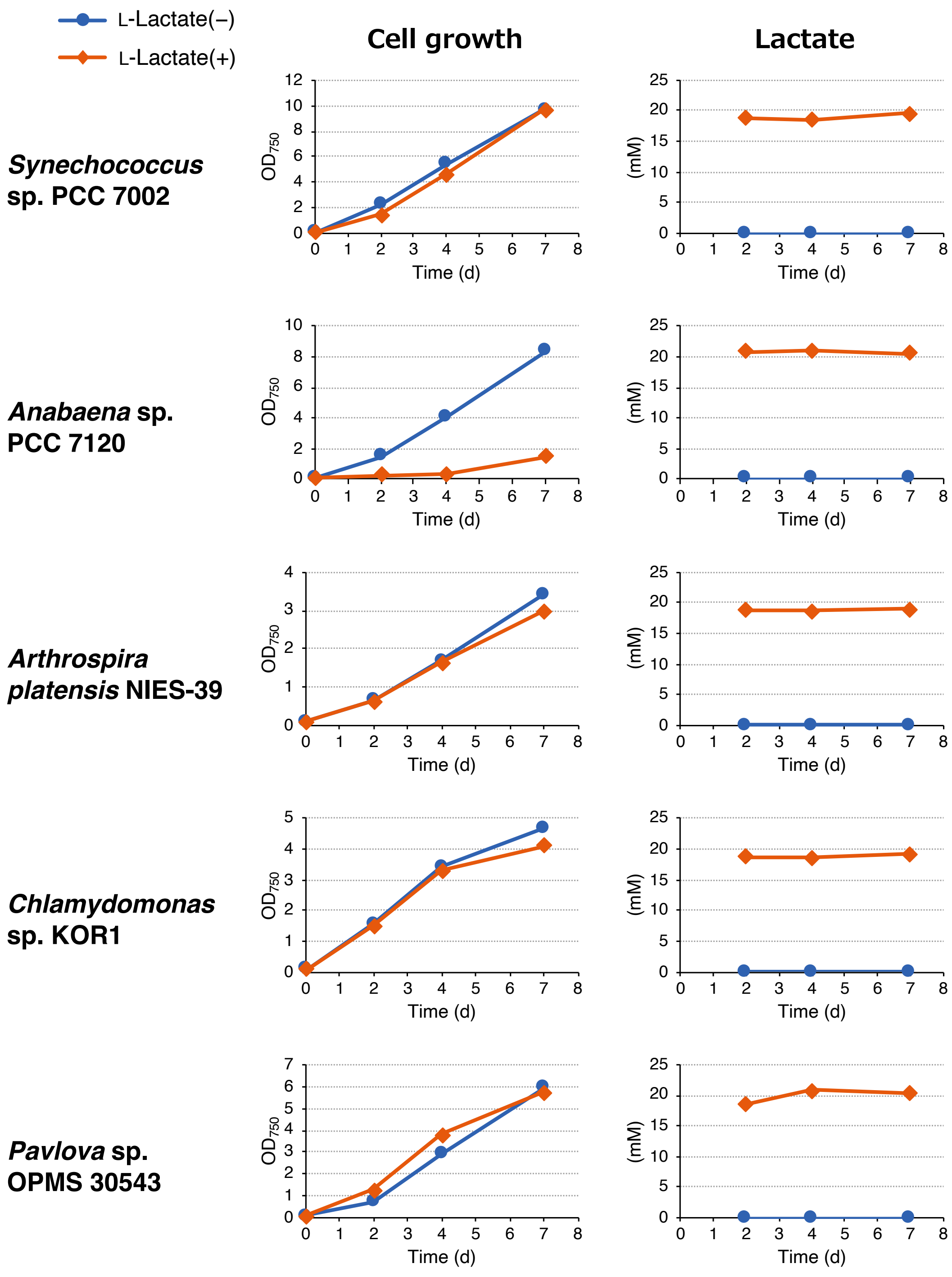

Supplementary Figure 2. Cultivation of cyanobacteria and microalgae in the presence of L-lactate. The cyanobacteria *Synechococcus* sp. PCC 7002, *Anabaena* sp. PCC 7120, and *Arthrospira platensis* NIES-39 and the microalgae *Chlamydomonas* sp. KOR1 and *Pavlova* sp. OPMS 30543 were phototrophically cultured in the absence or presence of 20 mM L-lactate (N = 1).

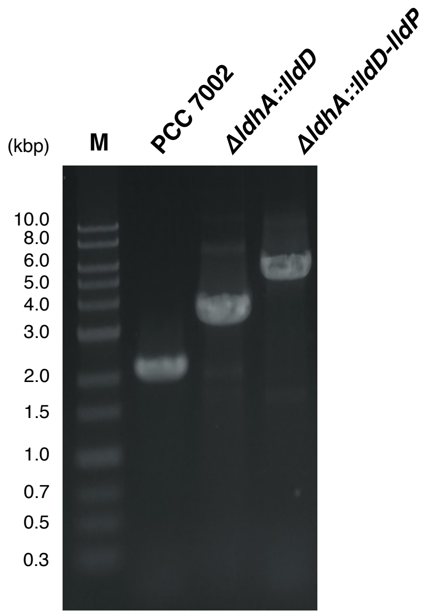

Supplementary Figure 3. Confirmation of integration and complete segregation by PCR. Integration of genetic elements (Fig. 3) into the pAQ7 plasmid of *Synechococcus* sp. PCC 7002 was confirmed using PCR. The following primer pairs were employed: 5′-CTATGACATGATTACGAATTCAGACATTTCCCACAGACCACATCAAATTA-3′ and 5′-CTGCAGGTCGACGGATCCCCGGGGGATCAATTTACGTCTTTGTTGGCGCA-3′. The expected product sizes were 2,031 bp for PCC 7002, 3,295 bp for *ΔldhA::lldD*, and 4,974 bp for *ΔldhA::lldD-lldP*.
